# Supplementary material for: Meta-Analysis of Mitochondrial DNA Control Region Diversity to Shed Light on Phylogenetic Relationship and Demographic History of African Sheep (Ovis aries) Breeds
Source: Biology (Basel). 2021 Aug 10;10(8):762. doi: 10.3390/biology10080762 (PMC8389696; doi:10.3390/biology10080762)
Supplement: Supplementary file 1 [file biology-10-00762-s001.zip › biology-1304585-supplementary-done (1).pdf]

biology

Table S1. A summary of studied sheep sequences and their respective NCBI GenBank accession identification numbers.

Region	Code	Breed	Accession ID	References
North Africa	ALG1-ALG7	Berbere	KU900012-KU900018	[1]
	ALG8-ALG24	Ouled Djellal	KU899996-KU900002 & MK601927 - MK601936	
	ALG25-ALG32	Rembi	KU900004 - KU900011	
	EGY1-EGY12	Rahmani	KC291221-KC291232	[2]
	EGY13-EGY24	Ossimi	KC291233-KC291244	
	EGY25-EGY34	Barki	KC291205 - KC291216	
	EGY35-EGY54	Sardi	KC461240 - KC461246	
	EGY55-EGY74	Romanov	DQ473858-DQ791219	
	MOR1-MOR32	Blanche	MN229116-MN229178	[3]
	MOR33-MOR63	Boujaad		
	MOR64-MOR91	Dman		
	MOR92-MOR125	Sardi		
	MOR126-MOR161	Timahdite		
	MOR162-MOR192	BeniGuil		
West Africa	WST1-WST44	West Africa	JN415706-JN415752	[4]
East Africa	KEN1-KEN3	Red Maasai	KT867683-KT867683	[5]
	KEN828-KEN906	Red Maasai	KT867725-KT867758	
	KEN753-KEN806	Black Head	KT867684-KT867713	
	KEN807-KEN824	East African fat tail	KT867714-KT867724	
	KEN911-KEN926	East African fat tail	KT867759- KT867768	
	KEN929-931	Dorper	KT867769-KT867771	
	TZ1-TZ3	Zanziberi	MK602124-MK602126	[6]
	SDN1-SDN18	Kabashi	MK602087-MK602100	[6]
	ETH1-ETH7	Afar	MK601947-MK601953	[6]
South Africa	SA1-SA10	South Africa Ancient	HM236334 - HM236322	[7]

Table S2. Population pairwise F_{ST} values.

	North Africa	East Africa	West Africa	South Africa
North Africa	0.000			
East Africa	0.098*	0.000		
West Africa	0.793*	0.619*	0.000	
South Africa	0.731*	0.596*	0.986*	0.000

* $p < 0.05$.

Table S3. Characteristics of the haplotypes.

Haplotype	Haplogroup	Sequences	Breed
Hap_1:	B	MOR12, MOR179	Blanche de Montagne, Beni-Guil
Hap_2:	B	MOR31	Blanche de Montagne
Hap_3:	B	MOR160	Timahdite
Hap_4:	B	MOR159, MOR87	Timahdite,D'man
Hap_5:	B	MOR175	Beni-Guil
Hap_6:	B	MOR150	Timahdite
Hap_7:	B	EGY56	Romanov
Hap_8:	B	MOR39	Boujaad
Hap_9:	B	EGY58	Romanov

Hap_10:	B	MOR83	D'man
Hap_11:	B	MOR106	Sardi
Hap_12:	B	MOR113, MOR41, MOR94, MOR33, KEN845, KEN845, KEN795, EGY22, EGY22, EGY24	Sardi, Boujaad, Sardi, Boujaad, Red maasai, Black head, Ossimi,Ossimi
Hap_13:	B	MOR119	Sardi
Hap_14:	B	MOR79	D'Man
Hap_15:	B	MOR178	Beni-Guil
Hap_16:	B	KEN843	Red maasai
Hap_17:	B	MOR147	Timahdite
Hap_18:	B	MOR65, MOR191	D'Man,Beni-Guil
Hap_19:	B	MOR93	Sardi
Hap_20:	B	MOR126	Timahdite
Hap_21:	B	MOR173	Beni-Guil
Hap_22:	B	MOR6	Blanche de Montagne
Hap_23:	B	MOR11	Blanche de Montagne
Hap_24:	B	MOR116, MOR122	Sardi, Sardi
Hap_25:	B	MOR52	Boujaad
Hap_26:	B	MOR4	Blanche de Montagne
Hap_27:	B	MOR46	Boujaad
Hap_28:	B	MOR162	Beni-Guil
Hap_29:	B	MOR111, MOR117	Sardi, Sardi
Hap_30:	B	MOR136	Timahdite
Hap_31:	B	MOR34	Boujaad
Hap_32:	B	MOR112	Sardi
Hap_33:	B	MOR185	Beni-Guil
Hap_34:	B	MOR180, MOR77	Beni-Guil,D'Man
Hap_35:	B	MOR0	Blanche de Montagne
Hap_36:	B	MOR71, MOR102	D'Man,Sardi
Hap_37:	B	MOR59	Boujaad
Hap_38:	B	MOR176, MOR78	Beni-Guil, D'Man
Hap_39:	B	MOR68, MOR70, MOR30, MOR91, MOR157, MOR161	D'Man, D'Man, Blanche de Montagne, D'Man, Timahdite, Timahdite
Hap_40:	B	MOR67	D'Man
Hap_41:	B	MOR118	Sardi
Hap_42:	B	MOR143	Timahdite
Hap_43:	B	MOR88	D'Man
Hap_44:	B	MOR76	D'Man
Hap_45:	B	MOR154	Timahdite
Hap_46:	B	MOR182	Beni-Guil
Hap_47:	B	MOR189	Beni-Guil
Hap_48:	B	MOR81	D'Man
Hap_49:	B	MOR42	Boujaad
Hap_50:	B	MOR153	Timahdite
Hap_51:	B	MOR8, MOR138, MOR184,MOR36, EGY31	Blanche de Montagne, Timahdite,Beni-Guil, Barki
Hap_52:	B	MOR20, MOR75	Blanche de Montagne, D'Man
Hap_53:	B	MOR109	sardi
Hap_54:	B	MOR35	Boujaad

Hap_55:	B	MOR186	Beni-Guil
Hap_56:	B	MOR128	Timahdite
Hap_57:	B	MOR141	Timahdite
Hap_58:	B	MOR48	Boujaad
Hap_59:	B	MOR142	Timahdite
Hap_60:	B	MOR18	Blanche de Montagne
Hap_61:	B	MOR69	D’Man
Hap_62:	B	MOR23, MOR25, MOR27,MOR29, MOR145	Blanche de Montagne,Blanche deMontagne,Blanche de Montagne,Blanche de Montagne,Timahdite
Hap_63:	B	MOR14	Blanche de Montagne
Hap_64:	B	MOR51	Boujaad
Hap_65:	C	MOR53	Boujaad
Hap_66:	C	MOR55	Boujaad
Hap_67:	C	MOR57	Boujaad
Hap_68:	C	MOR43	Boujaad
Hap_69:	B	MOR99, MOR97	Sardi,Sardi
Hap_70:	B	MOR16	Blanche de Montagne
Hap_71:	B	EGY55	Romanov
Hap_72:	B	KEN859	Red maasai
Hap_73:	B	MOR96	Sardi
Hap_74:	B	MOR167	Beni-Guil
Hap_75:	B	MOR104	Sardi
Hap_76:	B	MOR49	Boujaad
Hap_77:	B	MOR129	Timahdite
Hap_78:	B	MOR2	Blanche de Montagne
Hap_79:	B	MOR137	Timahdite
Hap_80:	B	MOR63	Boujaad
Hap_81:	B	MOR144	Timahdite
Hap_82:	B	MOR124	Sardi
Hap_83:	B	MOR100	Sardi
Hap_84:	B	MOR114	Sardi
Hap_85:	B	MOR64, MOR66, MOR84,MOR108	D’Man.D’Man,D’Man,Sardi
Hap_86:	B	MOR132	Timahdite
Hap_87:	B	MOR190, MOR90	Beni-Guil,D’Man
Hap_88:	B	MOR107	Sardi
Hap_89:	B	MOR3	Blanche de Montagne
Hap_90:	B	MOR1	Blanche de Montagne
Hap_91:	B	MOR9	Blanche de Montagne
Hap_92:	B	MOR44	Boujaad
Hap_93:	B	MOR115, MOR187	Sardi,Beni-Guil
Hap_94:	B	MOR19, MOR21, MOR17,MOR164,ALG7	Blanche de Montagne,Blanche deMontagne,Blanche de Montagne,Beni-Guil,Berbere
Hap_95:	B	MOR47	Boujaad
Hap_96:	B	MOR62	D’Man
Hap_97:	B	EGY57	Romanov
Hap_98:	B	MOR38	Boujaad
Hap_99:	B	MOR40	Boujaad

Hap_100:	B	MOR103	Sardi
Hap_101:	B	MOR125, MOR188	Sardi,Timahdite
Hap_102:	B	MOR56	Boujaad
Hap_103:	B	MOR139	Timahdite
Hap_104:	B	MOR130	Timahdite
Hap_105:	B	MOR140, MOR149	Timahdite,Timahdite
Hap_106:	B	MOR50	Boujaad
Hap_107:	B	MOR37	Boujaad
Hap_108:	B	MOR101	Sardi
Hap_109:	B	MOR45	Boujaad
Hap_110:	B	MOR151	Timahdite
Hap_111:	B	MOR121	Sardi
Hap_112:	B	MOR168	Beni-Guil
Hap_113:	B	MOR95	Sardi
Hap_114:	B	MOR155,KEN828	Timahdite,Red Maasai
Hap_115:	B	MOR146	Timahdite
Hap_116:	B	MOR98	Sardi
Hap_117:	B	KEN2,EGY47	Red maasai,Sardi
Hap_118:	B	KEN831	Red Maasai
Hap_119:	B	KEN793,KEN794	Black head, Black head
Hap_120:	B	KEN806	Black head
Hap_121:	B	KEN765	Black head
Hap_122:	B	KEN878	Red maasai
Hap_123:	B	KEN792, KEN797, KEN805,KEN888, KEN802	Black head, Black head, RedMaasai, Black head
Hap_124:	B	KEN847	Red Maasai
Hap_125:	B	KEN846	Red Maasai
Hap_126:	B	KEN886	Red Maasai
Hap_127:	B	KEN821, KEN834	East African fat tail, Red Maasai
Hap_128:	B	KEN861	Red Maasai
Hap_129:	B	KEN929	Dorper
Hap_130:	B	KEN815	East African fat tail
Hap_131:	B	KEN911	East African fat tail
Hap_132:	B	KEN755	Black head
Hap_133:	B	ALG28	Rembi
Hap_134:	B	KEN823	East African fat tail
Hap_135:	B	KEN863	Red Maasai
Hap_136:	B	KEN901, ALG10	Red Maasai
Hap_137:	B	KEN925	Dorper
Hap_138:	B	KEN889	Red Maasai
Hap_139:	B	KEN759	Black Head
Hap_140:	B	KEN3, KEN906	Red Maasai, Red Maasai
Hap_141:	B	KEN856, KEN923	Red Maasai, East African fat tail
Hap_142:	B	KEN869	Red Maasai
Hap_143:	B	KEN931	Dorper
Hap_144:	B	KEN899	Red Maasai
Hap_145:	B	KEN890	Red Maasai
Hap_146:	B	KEN798, KEN804, KEN799,KEN801	Back head, Black head, Blackhead, Black head

Hap_147:	B	KEN848	Red Maasai
Hap_148:	B	KEN808	East African fat tail
Hap_149:	B	KEN761	Black head
Hap_150:	B	KEN873	Red Maasai
Hap_151:	B	KEN807	East African fat tail
Hap_152:	B	KEN811, KEN812	East African fat tail,East Africanfat tail
Hap_153:	B	KEN851	Red Maasai
Hap_154:	B	KEN930	Dorper
Hap_155:	B	KEN921	East African fat tail
Hap_156:	B	KEN893	Red Maasai
Hap_157:	B	KEN920, KEN926, KEN882	East African fat tail, East Africanfat tail, Red Maasai
Hap_158:	B	KEN924	East African fat tail
Hap_159:	B	KEN904	Red Maasai
Hap_160:	B	KEN809	East African fat tail
Hap_161:	B	KEN919, KEN917	East African fat tail, East Africanfat tail
Hap_162:	B	KEN790	Black head
Hap_163:	B	KEN836	Red Maasai
Hap_164:	B	ALG5	Berbere
Hap_165:	B	ALG27	Rembi
Hap_166:	B	ALG11, ALG14	Ouled Djella, Ouled Djella
Hap_167:	B	ALG15	Ouled Djella
Hap_168:	B	ALG25	Rembi
Hap_169:	B	ALG3	Berbere
Hap_170:	B	ALG8	Ouled Djella
Hap_171:	B	ALG29	Rembi
Hap_172:	B	ALG32	Barki
Hap_173:	B	ALG2	Berbere
Hap_174:	B	ALG31	Rembi
Hap_175:	B	ALG6	Berbere
Hap_176:	B	ALG9	Ouled Djella
Hap_177:	C	ALG12	Ouled Djella
Hap_178:	C	ALG13	Ouled Djella
Hap_179:	B	EGY1	Rahmani
Hap_180:	B	EGY42	Sardi
Hap_181:	B	EGY2	Rahmani
Hap_182:	B	EGY6	Rahmani
Hap_183:	B	EGY9	Rahmani
Hap_184:	B	EGY11	Rahmani
Hap_185:	B	EGY3, EGY8	Rahmani,Rahmani
Hap_186:	C	EGY26	Barki
Hap_187:	B	EGY46	Sardi
Hap_188:	B	EGY29	Barki
Hap_189:	B	EGY17	Ouled Djella
Hap_190:	A	EGY32	Barki
Hap_191:	C	EGY34	Barki
Hap_192:	C	EGY23	Ossimi
Hap_193:	C	EGY13	Ossimi
Hap_194:	B	EGY16	Ossimi

Hap_195:	B	EGY50	Sardi
Hap_196:	B	EGY37	Sardi
Hap_197:	B	EGY4	Rahmani
Hap_198:	B	EGY54	Sardi
Hap_199:	B	EGY27	Barki
Hap_200:	B	EGY14	Ossimi
Hap_201:	B	EGY19	Ossimi
Hap_202:	B	EGY44	Sardi
Hap_203:	B	EGY10	Rahmani
Hap_204:	B	EGY21	Ossimi
Hap_205:	B	EGY7	Rahmani
Hap_206:	A	EGY30	Barki
Hap_207:	B	EGY39	Sardi
Hap_208:	B	EGY41	Sardi
Hap_209:	A	EGY25	Barki
Hap_210:	A	EGY33	Barki
Hap_211:	B	EGY15	Ossimi
Hap_212:	B	EGY5	Rahmani
Hap_213:	B	EGY28	Barki
Hap_214:	B	EGY38	Sardi
Hap_215:	B	EGY53	Sardi
Hap_216:	B	EGY51	Sardi
Hap_217:	B	EGY40	Sardi
Hap_218:	B	EGY48	Sardi
Hap_219:	B	EGY52	Sardi
Hap_220:	B	EGY43	Sardi
Hap_221:	B	EGY18	Ossimi
Hap_222:	B	EGY45	Sardi
Hap_223:	B	EGY20	Ossimi
Hap_224:	B	EGY49	Sardi
Hap_225:	B	SA2	South African ancient
Hap_226:	B	SA8	South African ancient
Hap_227:	B	SA3, SA13	South African ancient, SouthAfrican ancient
Hap_228:	B	SA4, SA5	South African ancient, SouthAfrican ancient
Hap_229:	B	SA7	South African ancient
Hap_230:	B	SA9	South African ancient
Hap_231:	B	SA12	South African ancient
Hap_232:	B	SA14	South African ancient
Hap_233:	B	ALG24, ETH7, ETH2, ETH1, SDN11, SDN13, SDN15, SDN17,SDN1, SDN7, SDN4, SDN12, TZ3, WST42, WST2, WST12, WST4, WST20, WST21, WST39, WST34, KEN918, KEN918, KEN918, KEN918, KEN756, KEN839, EGY69, EGY69, EGY64, EGY68	Ouled Djella, Afar, Afar, Afar, Kabashi, Kabasi, Kabashi, Kabashi, Kabashi, Kabashi, kabashi, kabashi, Zanziberi, WestAfrican, West African, West African, West African, West African, West African, West Africa, West Africa, East African fat tail, Back head, Red Maasai,Romanov, Romanov
Hap_234:	B	ALG21, WST8, WST28, WST37, EGY62	Oulled Djella, West Africa, West Africa, West Africa, Romanov

Hap_235:	B	SDN18, ALG19, WST36, WST5,WST11	Kabashi, Ouled Djella, West Africa, West Africa, West Africa
Hap_236:	B	WST35	West Africa
Hap_237:	B	WST22	West Africa
Hap_238:	B	WST18	West Africa
Hap_239:	B	SDN3, KEN837, KEN841	Kabashi,Red Maasai, Red Maasai
Hap_240:	B	SDN8, WST19, WST9, WST15, KEN824, KEN842, EGY74	Kabashi, West Africa, West Africa, West Africa, East African fat tail,Red Maasai, Romanov
Hap_241:	B	ALG18	Ouled Dlella
Hap_242:	B	SDN6	Kabashi
Hap_243:	B	ALG23, SDN10	Ouled Djella, Kabashi
Hap_244:	B	ALG17, WST1, WST44, WST23	Ouled Djella, West Africa, WestAfrica, West Africa
Hap_245:	B	ALG20, EGY70	Ouled Djella, Romanov
Hap_246:	B	ETH3, WST3	Afar, West Africa
Hap_247:	B	ETH4	Afar
Hap_248:	B	WST41	West Africa
Hap_249:	B	WST10, WST38	West Africa, West Africa
Hap_250:	B	WST17, WST32	West Africa, West Africa
Hap_251:	B	ETH6, WST30	Afar, West Africa
Hap_252:	B	ETH5	Afar
Hap_253:	B	ALG16, WST16, WST14, WST33	Ouled Djella, West Africa, WestAfrica, West Africa
Hap_254:	B	WST6	West Africa
Hap_255:	B	WST13	West Africa
Hap_256:	B	WST43	West Africa
Hap_257:	B	ALG22, WST26, WST27, WST25	Ouled Djella, West Africa, West Africa, West Africa
Hap_258:	B	TZ2	Zanziberi
Hap_259:	B	SDN9, SDN14, SDN16, TZ1, EGY59	Kabashi, Kabashi, Kabashi, Zanziberi, Romanov
Hap_260:	B	SDN2, WST24, WST29, EGY60	Kabashi, West Africa, West Africa,Ro-manov
Hap_261:	B	SDN5	Kabashi
Hap_262:	B	WST40	West Africa
Hap_263:	B	WST7	West Africa
Hap_264:	B	WST31	West Africa
Hap_265:	B	EGY66	Romanov
Hap_266:	B	EGY65	Romanov
Hap_267:	B	EGY67	Romanov
Hap_268:	B	EGY73	Romanov
Hap_269:	B	EGY71	Romanov
Hap_270:	B	EGY72	Romanov
Hap_271:	B	EGY63	Romanov
Hap_272:	B	EGY61	Romanov

Figure S1. Mismatch distribution graphs of MtDNA CR sequences of African sheep breeds based on pairwise differences. The solid; red line represents the expected distribution under expansion, the black bars represent the observed distribution under expansion.1, North African; 2, West Africa; 3, East Africa; 4, South Africa; 5, All sequences pooled together; Black bars: observed, red continuous line: expected mismatch distribution.

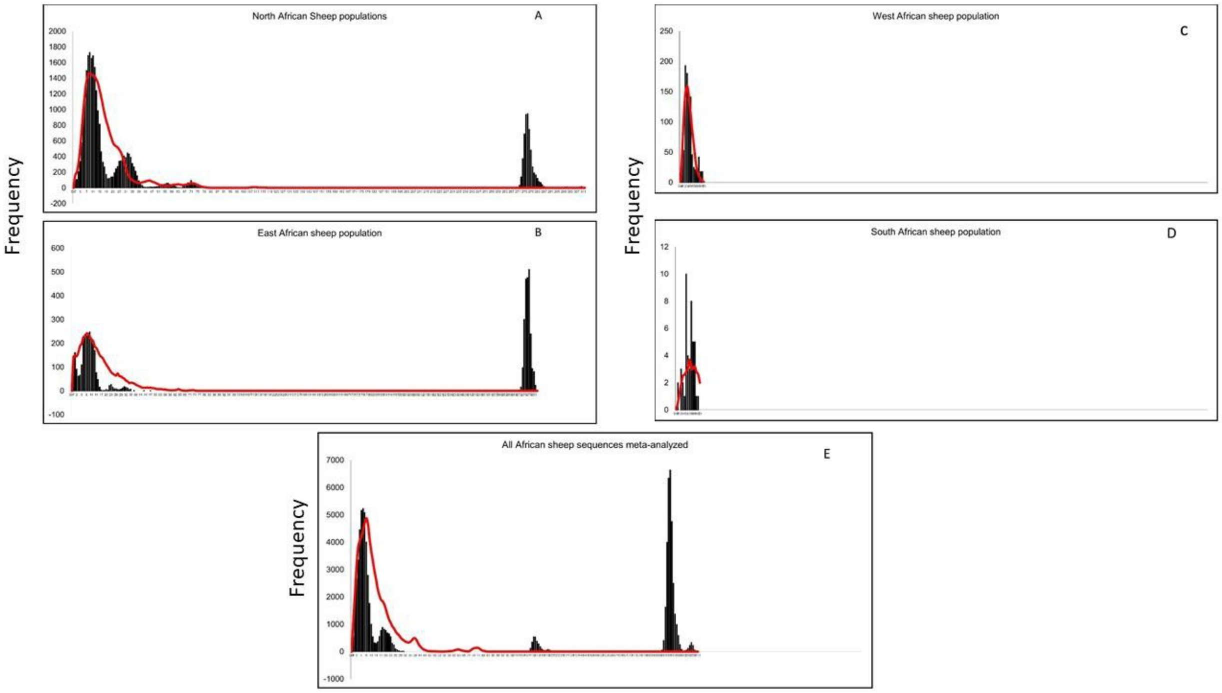

References

- Ghernouti, N., Bodinier, M., Ranebi, D., Maftah, A., Petit, D. and Gaouar, S.B.S. Control Region of mtDNA identifies three migration events of sheep breeds in Algeria. *Small Ruminant Research*, **2017**, *155*, 66-71.
- Othman, O.E., Balabel, E.A. and Abdel-Samad, M.F. Mitochondrial DNA diversity in five Egyptian sheep breeds. *Global Veterinaria*, **2014**, *12*, 369-375.
- Kandoussi, A., Boujenane, I., Auger, C., Serranito, B., Germot, A., Piro, M., Maftah, A., Badaoui, B. and Petit, D. The origin of sheep settlement in Western Mediterranean. *Scientific reports*, **2020**, *10,* 1-11.
- Revelo, H.A., López-Alvarez, D., Landi, V., Rizzo, L. and Alvarez, L.A. Mitochondrial DNA Variations in Colombian Creole Sheep Confirm an Iberian Origin and Shed Light on the Dynamics of Introduction Events of African Genotypes. *Animals*, **2020**, *10*, .1594.
- Resende, A., Gonçalves, J., Muigai, A.W. and Pereira, F. Mitochondrial DNA variation of domestic sheep (Ovis aries) in Kenya. *Animal genetics*, **2016**, *47*, 377-381.
- Al-Araimi,N.A. Jr., Al-Atiyat,R.M., Amir,N., Luzuriaga-Neira,A., Costa,V., Eisa,M.O., Mahgoub,O., Benaissa,M.H., Alfaris,A.A., Aljumaah,R.S., Elnakhla,S.M., Salem,M.M.I., El Khasmi,M., Ishag,I.A. and Beja-Pereira A. Maternal genetic variation of Arabian sheep reveals its origin and networking with African and Asian sheep. *Univ Porto, Res Cent Biodivers Genet* Resour (CIBIO), R Padre Arman Quintas 7, Vairao, Porto 4485-661, Port. **2019**.
- Horsburgh, K.A. and Rhines, A. Genetic characterization of an archaeological sheep assemblage from South Africa's Western Cape. Journal of Archaeological Science, **2010**, 37, 2906-2910.
